# 2′,6′-Bis(4-carb­oxy­phen­yl)-4,4′-bipyridin-1-ium nitrate 0.25-hydrate

**DOI:** 10.1107/S1600536811051506

**Published:** 2011-12-07

**Authors:** Yaping Li, Hu Zang, Guanfang Su

**Affiliations:** a Department of Ophthalmology, The Second Hospital of Jilin University, Changchun 130041, People’s Republic of China; b Department of Orthopedics, The China–Japan Union Hospital of Jilin University, Changchun 130033, People’s Republic of China

## Abstract

In the title compound, C_24_H_17_N_2_O_4_^+^·NO_3_^−^·0.25H_2_O, the central pyridine ring of the 2′,6′-bis­(4-carb­oxy­phen­yl)-4,4′-bipyridin-1-ium cation is almost coplanar with one benzene ring [dihedral angle = 1.03 (5)°], while it makes dihedral angles of 9.59 (5)° with the other benzene ring and 13.66 (6)° with the pyridinium ring. In the crystal, N—H⋯O and O—H⋯O hydrogen bonds link the cations and nitrate anions into a sheet in the (302) plane. The crystal structure also exhibits π–π inter­actions between the central pyridine ring and the benzene rings of neighboring mol­ecules [centroid–centroid distance = 3.6756 (13) Å].

## Related literature

For the properties and applications of mol­ecular-ionic crystals, see: Katrusiak & Szafrański (2006 ▶); Liao *et al.* (2008 ▶); Wang (2010 ▶).
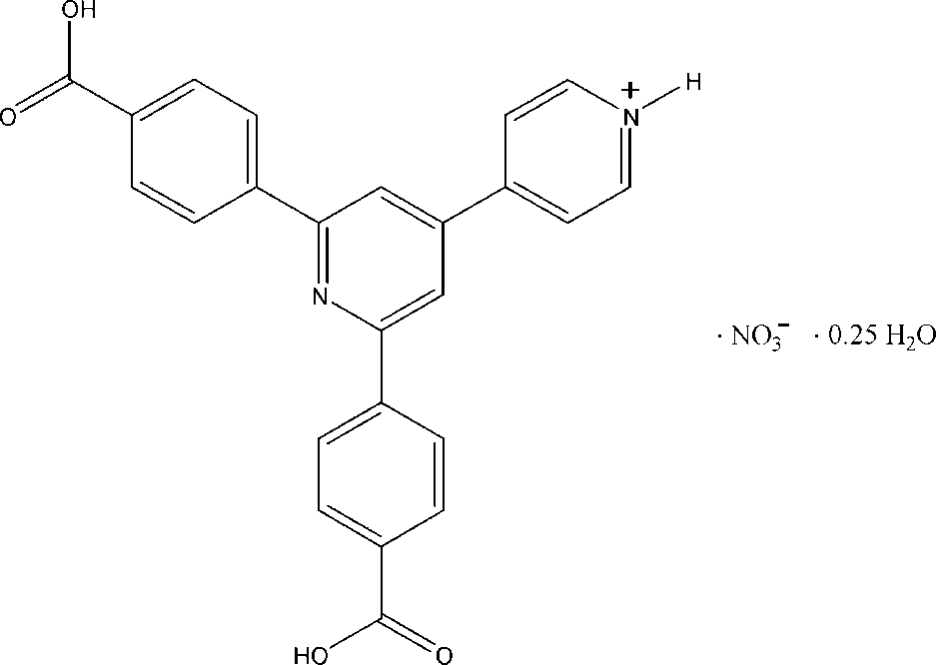


## Experimental

### 

#### Crystal data

C_24_H_17_N_2_O_4_^+^·NO_3_^−^·0.25H_2_O
*M* *_r_* = 463.91
Monoclinic, 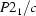

*a* = 13.6978 (11) Å
*b* = 16.0688 (12) Å
*c* = 9.6354 (8) Å
β = 92.696 (1)°
*V* = 2118.5 (3) Å^3^
*Z* = 4
Mo *K*α radiation
μ = 0.11 mm^−1^
*T* = 293 K
0.25 × 0.21 × 0.20 mm

#### Data collection

Bruker APEXII CCD diffractometer
Absorption correction: multi-scan (*SADABS*; Bruker, 2001 ▶) *T*_min_ = 0.973, *T*_max_ = 0.978
11529 measured reflections
4158 independent reflections
2605 reflections with *I* > 2σ(*I*)
*R*_int_ = 0.040

#### Refinement

*R*[*F* ^2^ > 2σ(*F* ^2^)] = 0.050
*wR*(*F* ^2^) = 0.150
*S* = 1.04
4158 reflections
316 parameters
H-atom parameters constrained
Δρ_max_ = 0.30 e Å^−3^
Δρ_min_ = −0.27 e Å^−3^

### 

Data collection: *APEX2* (Bruker, 2007 ▶); cell refinement: *SAINT* (Bruker, 2007 ▶); data reduction: *SAINT*; program(s) used to solve structure: *SHELXTL* (Sheldrick, 2008 ▶); program(s) used to refine structure: *SHELXTL*; molecular graphics: *XP* in *SHELXTL* and *Mercury* (Macrae *et al.*, 2006 ▶); software used to prepare material for publication: *SHELXTL*.

## Supplementary Material

Crystal structure: contains datablock(s) global, I. DOI: 10.1107/S1600536811051506/bt5723sup1.cif

Structure factors: contains datablock(s) I. DOI: 10.1107/S1600536811051506/bt5723Isup2.hkl

Supplementary material file. DOI: 10.1107/S1600536811051506/bt5723Isup3.cml

Additional supplementary materials:  crystallographic information; 3D view; checkCIF report

## Figures and Tables

**Table 1:** Hydrogen-bond geometry (Å, °)

*D*—H⋯*A*	*D*—H	H⋯*A*	*D*⋯*A*	*D*—H⋯*A*
N1—H1⋯O5^i^	0.86	1.84	2.697 (2)	171
O2—H2*A*⋯O3^ii^	0.82	1.93	2.728 (2)	163
O4—H4⋯O5	0.82	1.78	2.598 (2)	173
O1*W*—H1*A*⋯O1*W*^iii^	0.92	2.14	2.963 (18)	149
O1*W*—H1*B*⋯O1^iv^	0.90	2.22	3.059 (8)	154

Symmetry codes: (i) 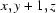; (ii) 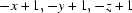; (iii) 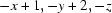; (iv) 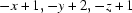.

**Fig. 1.**
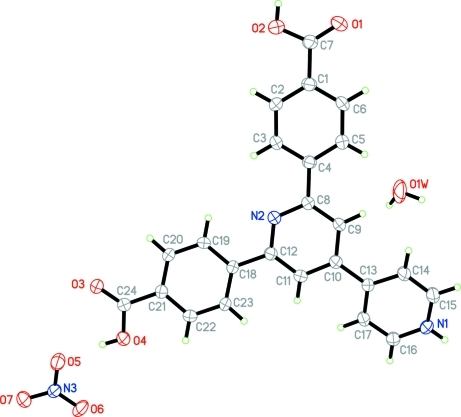
The molecular structure of the title compound. Displacement ellipsoids are drawn at the 50% probability level.

**Fig. 2.**
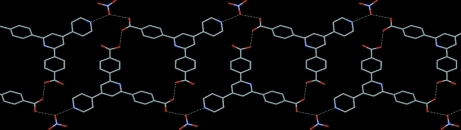
View of the one-dimensional ribbon structure of the title compound built by hydrogen bonds (dashed lines).

## supplementary crystallographic information

### Comment

Recently much attention has been devoted to simple molecular-ionic crystals containing organic cations and anions due to the tunability of their special structural features and their interesting physical properties (Katrusiak & Szafrański, 2006; Wang, 2010). Molecular building blocks associated with pyridine carboxylic acids are widely used in chiral catalysis, photoelectronic materials, hematopathology and medicine (Liao *et al.*, 2008). In our laboratory, the compound containing a 2,6-bis(4-carboxyphenyl)-4,4'-bipyridinium cation, an NO_3_^-^ anion and quarter water molecules has been synthesized and its crystal structure is reported here.

In the title compound (Fig. 1), the central pyridine ring of the cation is almost coplanar with one benzene ring, making a dihedral angle of 1.03 (5)°, while it makes dihedral angles of 9.59 (5)° with the other benzene ring and 13.66 (6)° with the protonated pyridinium ring. N—H···O and O—H···O hydrogen bonds link the organic cations and nitrate anions into a one-dimensional ribbon along [0 1 0] (Fig. 2). The crystal structure also exhibits π–π interactions between the central pyridine rings and the benzene rings of neighboring molecules [centroid–centroid distance = 3.6756 (13) Å].

### Experimental

A mixture of 2,6-di-4-carboxyphenyl-4,4'-bipyridine (0.2 mmol, 0.080 g), nitric acid (0.5 ml, 6 mol/*L*), ethanol (10 ml) and H_2_O (5 ml) was stirred for 10 min. A small amount of white precipitate was filtered off and the filtrate was kept for several days at ambient conditions. Colorless block crystals of the title compound were isolated.

### Refinement

H atoms on C and N atoms were positioned geometrically and refined as riding atoms, with C—H = 0.93 and N—H = 0.86 Å and with *U*_iso_(H) = 1.2*U*_eq_(C, N). According to the electron density and element analysis, the site occupation factor of the water molecule   was fixed at 0.25. H atoms of the water molecule were set to an arbitrary postion and refined as riding atoms, with *U*_iso_(H) = 1.5*U*_eq_(O).

### Crystal data

**Table:**

C_24_H_17_N_2_O_4_^+^·NO_3_^−^·0.25H_2_O	*F*(000) = 962
*M**_r_* = 463.91	*D*_x_ = 1.454 Mg m^−^^3^
Monoclinic, *P*2_1_/*c*	Mo *K*α radiation, λ = 0.71073 Å
Hall symbol: -P 2ybc	Cell parameters from 4158 reflections
*a* = 13.6978 (11) Å	θ = 1.0–26.0°
*b* = 16.0688 (12) Å	µ = 0.11 mm^−^^1^
*c* = 9.6354 (8) Å	*T* = 293 K
β = 92.696 (1)°	Block, colorless
*V* = 2118.5 (3) Å^3^	0.25 × 0.21 × 0.20 mm
*Z* = 4	

### Data collection

**Table:**

Bruker APEXII CCD diffractometer	4158 independent reflections
Radiation source: fine-focus sealed tube	2605 reflections with *I* > 2σ(*I*)
graphite	*R*_int_ = 0.040
φ and ω scans	θ_max_ = 26.0°, θ_min_ = 2.0°
Absorption correction: multi-scan (*SADABS*; Bruker, 2001)	*h* = −16→16
*T*_min_ = 0.973, *T*_max_ = 0.978	*k* = −19→17
11529 measured reflections	*l* = −9→11

### Refinement

**Table:**

Refinement on *F*^2^	Primary atom site location: structure-invariant direct methods
Least-squares matrix: full	Secondary atom site location: difference Fourier map
*R*[*F*^2^ > 2σ(*F*^2^)] = 0.050	Hydrogen site location: inferred from neighbouring sites
*wR*(*F*^2^) = 0.150	H-atom parameters constrained
*S* = 1.04	*w* = 1/[σ^2^(*F*_o_^2^) + (0.0724*P*)^2^ + 0.0957*P*] where *P* = (*F*_o_^2^ + 2*F*_c_^2^)/3
4158 reflections	(Δ/σ)_max_ < 0.001
316 parameters	Δρ_max_ = 0.30 e Å^−^^3^
0 restraints	Δρ_min_ = −0.27 e Å^−^^3^

### Special details

**Table:**

Geometry. All e.s.d.'s (except the e.s.d. in the dihedral angle between two l.s. planes) are estimated using the full covariance matrix. The cell e.s.d.'s are taken into account individually in the estimation of e.s.d.'s in distances, angles and torsion angles; correlations between e.s.d.'s in cell parameters are only used when they are defined by crystal symmetry. An approximate (isotropic) treatment of cell e.s.d.'s is used for estimating e.s.d.'s involving l.s. planes.
Refinement. Refinement of *F*^2^ against ALL reflections. The weighted *R*-factor *wR* and goodness of fit *S* are based on *F*^2^, conventional *R*-factors *R* are based on *F*, with *F* set to zero for negative *F*^2^. The threshold expression of *F*^2^ > σ(*F*^2^) is used only for calculating *R*-factors(gt) *etc*. and is not relevant to the choice of reflections for refinement. *R*-factors based on *F*^2^ are statistically about twice as large as those based on *F*, and *R*- factors based on ALL data will be even larger.

### Fractional atomic coordinates and isotropic or equivalent isotropic displacement parameters (Å^2^)

**Table:**

	*x*	*y*	*z*	*U*_iso_*/*U*_eq_	Occ. (<1)
C1	0.42765 (16)	0.78749 (14)	0.5913 (2)	0.0286 (5)	
C2	0.46419 (18)	0.71363 (14)	0.5406 (3)	0.0334 (6)	
H2	0.4404	0.6631	0.5715	0.040*	
C3	0.53576 (18)	0.71478 (14)	0.4445 (3)	0.0337 (6)	
H3	0.5597	0.6647	0.4117	0.040*	
C4	0.57286 (16)	0.78937 (13)	0.3957 (2)	0.0258 (5)	
C5	0.53495 (17)	0.86287 (14)	0.4469 (3)	0.0330 (6)	
H5	0.5579	0.9135	0.4152	0.040*	
C6	0.46397 (17)	0.86229 (14)	0.5438 (2)	0.0322 (6)	
H6	0.4404	0.9123	0.5774	0.039*	
C7	0.35134 (17)	0.78863 (14)	0.6971 (3)	0.0321 (6)	
C8	0.65044 (16)	0.78741 (13)	0.2920 (2)	0.0254 (5)	
C9	0.69124 (16)	0.85873 (14)	0.2365 (2)	0.0279 (5)	
H9	0.6711	0.9110	0.2651	0.033*	
C10	0.76210 (16)	0.85180 (13)	0.1382 (2)	0.0260 (5)	
C11	0.78848 (16)	0.77208 (14)	0.0987 (2)	0.0273 (5)	
H11	0.8352	0.7648	0.0327	0.033*	
C12	0.74523 (16)	0.70341 (13)	0.1575 (2)	0.0249 (5)	
C13	0.80567 (16)	0.92684 (13)	0.0741 (2)	0.0261 (5)	
C14	0.7948 (2)	1.00598 (14)	0.1296 (3)	0.0438 (7)	
H14	0.7611	1.0128	0.2104	0.053*	
C15	0.8333 (2)	1.07395 (15)	0.0661 (3)	0.0460 (7)	
H15	0.8248	1.1267	0.1033	0.055*	
C16	0.89662 (18)	0.99033 (13)	−0.1044 (3)	0.0328 (6)	
H16	0.9319	0.9856	−0.1842	0.039*	
C17	0.85909 (17)	0.92067 (14)	−0.0450 (2)	0.0306 (6)	
H17	0.8693	0.8688	−0.0844	0.037*	
C18	0.76886 (16)	0.61666 (13)	0.1168 (2)	0.0247 (5)	
C19	0.71249 (16)	0.55022 (14)	0.1607 (2)	0.0295 (5)	
H19	0.6597	0.5606	0.2154	0.035*	
C20	0.73369 (16)	0.46961 (14)	0.1246 (2)	0.0303 (6)	
H20	0.6958	0.4262	0.1563	0.036*	
C21	0.81127 (16)	0.45261 (13)	0.0410 (2)	0.0272 (5)	
C22	0.86680 (18)	0.51865 (14)	−0.0048 (3)	0.0323 (6)	
H22	0.9182	0.5083	−0.0622	0.039*	
C23	0.84681 (16)	0.59942 (14)	0.0335 (2)	0.0301 (6)	
H23	0.8857	0.6427	0.0036	0.036*	
C24	0.83576 (16)	0.36588 (14)	0.0027 (2)	0.0286 (5)	
N1	0.88275 (14)	1.06493 (12)	−0.0482 (2)	0.0337 (5)	
H1	0.9064	1.1082	−0.0869	0.040*	
N2	0.67797 (13)	0.71123 (10)	0.25346 (19)	0.0260 (4)	
N3	0.99617 (14)	0.19689 (12)	−0.2573 (2)	0.0312 (5)	
O2	0.31962 (13)	0.71380 (10)	0.72797 (19)	0.0456 (5)	
H2A	0.2781	0.7176	0.7863	0.068*	
O1	0.32181 (13)	0.85207 (11)	0.74803 (19)	0.0469 (5)	
O4	0.89796 (12)	0.36227 (9)	−0.09689 (18)	0.0387 (5)	
H4	0.9094	0.3135	−0.1148	0.058*	
O3	0.80256 (12)	0.30430 (9)	0.05852 (18)	0.0350 (4)	
O5	0.94948 (15)	0.21008 (10)	−0.14753 (19)	0.0496 (5)	
O6	1.02518 (13)	0.25580 (11)	−0.32456 (19)	0.0451 (5)	
O7	1.01033 (13)	0.12364 (10)	−0.28914 (18)	0.0428 (5)	
O1W	0.5358 (6)	1.0131 (4)	0.1459 (10)	0.062 (2)	0.25
H1A	0.5359	0.9943	0.0556	0.092*	0.25
H1B	0.5683	1.0618	0.1548	0.092*	0.25

### Atomic displacement parameters (Å^2^)

**Table:**

	*U*^11^	*U*^22^	*U*^33^	*U*^12^	*U*^13^	*U*^23^
C1	0.0288 (13)	0.0303 (13)	0.0272 (13)	0.0037 (10)	0.0056 (10)	0.0019 (10)
C2	0.0407 (14)	0.0244 (13)	0.0365 (15)	−0.0024 (10)	0.0156 (12)	0.0025 (10)
C3	0.0403 (14)	0.0224 (12)	0.0398 (15)	0.0029 (10)	0.0149 (12)	−0.0015 (10)
C4	0.0257 (12)	0.0270 (12)	0.0248 (13)	0.0015 (9)	0.0037 (10)	−0.0004 (9)
C5	0.0345 (14)	0.0245 (13)	0.0407 (15)	0.0012 (10)	0.0095 (12)	0.0001 (11)
C6	0.0363 (14)	0.0243 (13)	0.0367 (15)	0.0064 (10)	0.0100 (11)	−0.0032 (10)
C7	0.0315 (13)	0.0303 (14)	0.0351 (15)	0.0043 (11)	0.0084 (11)	0.0037 (11)
C8	0.0256 (12)	0.0234 (12)	0.0274 (13)	0.0015 (9)	0.0030 (10)	−0.0001 (9)
C9	0.0318 (13)	0.0221 (12)	0.0305 (13)	0.0007 (10)	0.0091 (10)	−0.0036 (10)
C10	0.0278 (12)	0.0246 (12)	0.0259 (13)	0.0004 (10)	0.0038 (10)	−0.0009 (9)
C11	0.0257 (12)	0.0264 (12)	0.0304 (13)	0.0010 (10)	0.0072 (10)	−0.0011 (10)
C12	0.0256 (12)	0.0243 (12)	0.0249 (13)	0.0010 (9)	0.0024 (10)	0.0010 (9)
C13	0.0268 (12)	0.0213 (12)	0.0305 (13)	0.0003 (9)	0.0051 (10)	0.0005 (9)
C14	0.0612 (18)	0.0250 (14)	0.0480 (17)	−0.0023 (12)	0.0325 (14)	−0.0033 (11)
C15	0.0628 (18)	0.0240 (14)	0.0538 (18)	−0.0013 (12)	0.0293 (15)	−0.0022 (12)
C16	0.0391 (14)	0.0277 (13)	0.0327 (14)	−0.0011 (11)	0.0119 (12)	−0.0005 (11)
C17	0.0363 (13)	0.0225 (12)	0.0333 (14)	0.0019 (10)	0.0063 (11)	−0.0026 (10)
C18	0.0258 (12)	0.0208 (12)	0.0276 (13)	−0.0004 (9)	0.0033 (10)	0.0023 (9)
C19	0.0273 (12)	0.0264 (13)	0.0361 (14)	0.0012 (10)	0.0134 (10)	0.0006 (10)
C20	0.0305 (13)	0.0223 (12)	0.0389 (15)	−0.0031 (10)	0.0098 (11)	0.0020 (10)
C21	0.0290 (12)	0.0229 (12)	0.0300 (13)	−0.0015 (10)	0.0047 (10)	−0.0012 (10)
C22	0.0329 (13)	0.0294 (13)	0.0357 (14)	0.0005 (11)	0.0147 (11)	−0.0011 (11)
C23	0.0327 (13)	0.0200 (12)	0.0384 (14)	−0.0020 (10)	0.0121 (11)	0.0021 (10)
C24	0.0282 (13)	0.0260 (13)	0.0321 (14)	−0.0001 (10)	0.0057 (11)	0.0004 (10)
N1	0.0399 (12)	0.0244 (11)	0.0380 (12)	−0.0038 (9)	0.0146 (10)	0.0045 (9)
N2	0.0272 (10)	0.0231 (10)	0.0278 (11)	0.0014 (8)	0.0043 (8)	−0.0005 (8)
N3	0.0333 (11)	0.0273 (11)	0.0338 (12)	−0.0033 (9)	0.0089 (9)	0.0009 (9)
O2	0.0508 (11)	0.0341 (10)	0.0541 (13)	0.0027 (8)	0.0271 (10)	0.0052 (9)
O1	0.0550 (12)	0.0346 (10)	0.0534 (13)	0.0088 (9)	0.0277 (10)	−0.0016 (9)
O4	0.0485 (10)	0.0250 (9)	0.0446 (11)	0.0010 (8)	0.0218 (9)	−0.0032 (8)
O3	0.0389 (10)	0.0240 (9)	0.0431 (11)	−0.0011 (7)	0.0141 (8)	0.0018 (7)
O5	0.0812 (14)	0.0267 (10)	0.0441 (12)	−0.0023 (9)	0.0370 (11)	−0.0029 (8)
O6	0.0528 (12)	0.0370 (10)	0.0466 (12)	−0.0102 (9)	0.0156 (9)	0.0122 (9)
O7	0.0522 (12)	0.0295 (10)	0.0482 (12)	0.0052 (8)	0.0181 (9)	−0.0051 (8)
O1W	0.069 (5)	0.023 (4)	0.093 (7)	0.006 (4)	−0.002 (5)	0.010 (4)

### Geometric parameters (Å, °)

**Table:**

C1—C2	1.386 (3)	C15—N1	1.328 (3)
C1—C6	1.387 (3)	C15—H15	0.9300
C1—C7	1.495 (3)	C16—N1	1.333 (3)
C2—C3	1.380 (3)	C16—C17	1.369 (3)
C2—H2	0.9300	C16—H16	0.9300
C3—C4	1.392 (3)	C17—H17	0.9300
C3—H3	0.9300	C18—C23	1.393 (3)
C4—C5	1.390 (3)	C18—C19	1.395 (3)
C4—C8	1.493 (3)	C19—C20	1.376 (3)
C5—C6	1.379 (3)	C19—H19	0.9300
C5—H5	0.9300	C20—C21	1.390 (3)
C6—H6	0.9300	C20—H20	0.9300
C7—O1	1.209 (3)	C21—C22	1.390 (3)
C7—O2	1.317 (3)	C21—C24	1.484 (3)
C8—N2	1.338 (3)	C22—C23	1.380 (3)
C8—C9	1.393 (3)	C22—H22	0.9300
C9—C10	1.392 (3)	C23—H23	0.9300
C9—H9	0.9300	C24—O3	1.224 (3)
C10—C11	1.389 (3)	C24—O4	1.314 (3)
C10—C13	1.492 (3)	N1—H1	0.8600
C11—C12	1.386 (3)	N3—O6	1.224 (2)
C11—H11	0.9300	N3—O7	1.234 (2)
C12—N2	1.341 (3)	N3—O5	1.279 (2)
C12—C18	1.488 (3)	O2—H2A	0.8200
C13—C14	1.390 (3)	O4—H4	0.8200
C13—C17	1.393 (3)	O1W—H1A	0.9210
C14—C15	1.370 (3)	O1W—H1B	0.9027
C14—H14	0.9300		
			
C2—C1—C6	119.0 (2)	C13—C14—H14	119.8
C2—C1—C7	121.8 (2)	N1—C15—C14	120.4 (2)
C6—C1—C7	119.2 (2)	N1—C15—H15	119.8
C3—C2—C1	120.3 (2)	C14—C15—H15	119.8
C3—C2—H2	119.8	N1—C16—C17	120.1 (2)
C1—C2—H2	119.8	N1—C16—H16	119.9
C2—C3—C4	121.3 (2)	C17—C16—H16	119.9
C2—C3—H3	119.3	C16—C17—C13	120.6 (2)
C4—C3—H3	119.3	C16—C17—H17	119.7
C5—C4—C3	117.6 (2)	C13—C17—H17	119.7
C5—C4—C8	123.0 (2)	C23—C18—C19	118.2 (2)
C3—C4—C8	119.4 (2)	C23—C18—C12	121.34 (19)
C6—C5—C4	121.4 (2)	C19—C18—C12	120.5 (2)
C6—C5—H5	119.3	C20—C19—C18	121.2 (2)
C4—C5—H5	119.3	C20—C19—H19	119.4
C5—C6—C1	120.3 (2)	C18—C19—H19	119.4
C5—C6—H6	119.9	C19—C20—C21	120.5 (2)
C1—C6—H6	119.9	C19—C20—H20	119.7
O1—C7—O2	123.9 (2)	C21—C20—H20	119.7
O1—C7—C1	123.0 (2)	C22—C21—C20	118.6 (2)
O2—C7—C1	113.0 (2)	C22—C21—C24	120.3 (2)
N2—C8—C9	121.5 (2)	C20—C21—C24	121.1 (2)
N2—C8—C4	115.06 (19)	C23—C22—C21	120.9 (2)
C9—C8—C4	123.41 (19)	C23—C22—H22	119.5
C10—C9—C8	120.0 (2)	C21—C22—H22	119.5
C10—C9—H9	120.0	C22—C23—C18	120.5 (2)
C8—C9—H9	120.0	C22—C23—H23	119.7
C11—C10—C9	117.3 (2)	C18—C23—H23	119.7
C11—C10—C13	121.2 (2)	O3—C24—O4	123.5 (2)
C9—C10—C13	121.5 (2)	O3—C24—C21	123.8 (2)
C12—C11—C10	120.0 (2)	O4—C24—C21	112.65 (19)
C12—C11—H11	120.0	C15—N1—C16	121.6 (2)
C10—C11—H11	120.0	C15—N1—H1	119.2
N2—C12—C11	121.9 (2)	C16—N1—H1	119.2
N2—C12—C18	115.74 (19)	C8—N2—C12	119.23 (19)
C11—C12—C18	122.4 (2)	O6—N3—O7	123.2 (2)
C14—C13—C17	116.9 (2)	O6—N3—O5	119.81 (19)
C14—C13—C10	121.8 (2)	O7—N3—O5	117.02 (18)
C17—C13—C10	121.3 (2)	C7—O2—H2A	109.5
C15—C14—C13	120.5 (2)	C24—O4—H4	109.5
C15—C14—H14	119.8	H1A—O1W—H1B	110.5
			
C6—C1—C2—C3	0.1 (4)	C17—C13—C14—C15	1.6 (4)
C7—C1—C2—C3	−178.9 (2)	C10—C13—C14—C15	−178.2 (2)
C1—C2—C3—C4	−0.2 (4)	C13—C14—C15—N1	−0.9 (4)
C2—C3—C4—C5	−0.2 (4)	N1—C16—C17—C13	0.4 (4)
C2—C3—C4—C8	179.9 (2)	C14—C13—C17—C16	−1.4 (4)
C3—C4—C5—C6	0.7 (4)	C10—C13—C17—C16	178.4 (2)
C8—C4—C5—C6	−179.4 (2)	N2—C12—C18—C23	−170.8 (2)
C4—C5—C6—C1	−0.8 (4)	C11—C12—C18—C23	10.3 (3)
C2—C1—C6—C5	0.4 (4)	N2—C12—C18—C19	9.7 (3)
C7—C1—C6—C5	179.5 (2)	C11—C12—C18—C19	−169.2 (2)
C2—C1—C7—O1	176.2 (2)	C23—C18—C19—C20	0.8 (3)
C6—C1—C7—O1	−2.9 (4)	C12—C18—C19—C20	−179.7 (2)
C2—C1—C7—O2	−4.2 (4)	C18—C19—C20—C21	−1.1 (4)
C6—C1—C7—O2	176.7 (2)	C19—C20—C21—C22	0.1 (4)
C5—C4—C8—N2	−179.6 (2)	C19—C20—C21—C24	179.2 (2)
C3—C4—C8—N2	0.3 (3)	C20—C21—C22—C23	1.2 (4)
C5—C4—C8—C9	−0.5 (4)	C24—C21—C22—C23	−177.9 (2)
C3—C4—C8—C9	179.4 (2)	C21—C22—C23—C18	−1.5 (4)
N2—C8—C9—C10	0.3 (3)	C19—C18—C23—C22	0.4 (3)
C4—C8—C9—C10	−178.8 (2)	C12—C18—C23—C22	−179.1 (2)
C8—C9—C10—C11	0.6 (3)	C22—C21—C24—O3	166.1 (2)
C8—C9—C10—C13	178.5 (2)	C20—C21—C24—O3	−13.0 (4)
C9—C10—C11—C12	−0.5 (3)	C22—C21—C24—O4	−13.2 (3)
C13—C10—C11—C12	−178.5 (2)	C20—C21—C24—O4	167.7 (2)
C10—C11—C12—N2	−0.3 (3)	C14—C15—N1—C16	−0.1 (4)
C10—C11—C12—C18	178.5 (2)	C17—C16—N1—C15	0.4 (4)
C11—C10—C13—C14	−167.6 (2)	C9—C8—N2—C12	−1.1 (3)
C9—C10—C13—C14	14.5 (4)	C4—C8—N2—C12	178.05 (19)
C11—C10—C13—C17	12.6 (3)	C11—C12—N2—C8	1.2 (3)
C9—C10—C13—C17	−165.3 (2)	C18—C12—N2—C8	−177.77 (19)

### Hydrogen-bond geometry (Å, °)

**Table:**

*D*—H···*A*	*D*—H	H···*A*	*D*···*A*	*D*—H···*A*
N1—H1···O5^i^	0.86	1.84	2.697 (2)	171
O2—H2A···O3^ii^	0.82	1.93	2.728 (2)	163
O4—H4···O5	0.82	1.78	2.598 (2)	173
O1W—H1A···O1W^iii^	0.92	2.14	2.963 (18)	149
O1W—H1B···O1^iv^	0.90	2.22	3.059 (8)	154

Symmetry codes: (i) *x*, *y*+1, *z*; (ii) −*x*+1, −*y*+1, −*z*+1; (iii) −*x*+1, −*y*+2, −*z*; (iv) −*x*+1, −*y*+2, −*z*+1.
